# Severe Upper Airway Obstruction in a Patient With Infectious Mononucleosis

**DOI:** 10.7759/cureus.58735

**Published:** 2024-04-22

**Authors:** Diego P Peralta, Aymara Y Chang

**Affiliations:** 1 Infectious Diseases, El Paso VA Health Care System, El Paso, USA; 2 Internal Medicine, Texas Tech University Health Sciences Center El Paso, El Paso, USA

**Keywords:** waldeyer's tonsillar ring, upper airway obstruction, corticosteroids, infectious mononucleosis, epstein-barr virus

## Abstract

Infectious mononucleosis (IM) is a clinical disease caused by the Epstein-Barr virus (EBV). Common presenting symptoms include sore throat, lymph node enlargement, fever, and malaise. Although severe upper airway obstruction is uncommon, it is a potentially fatal complication that requires immediate intervention. We describe the case of an 18-year-old Hispanic man who presented with a progressive sore throat and difficulty speaking, requiring endotracheal intubation for airway protection. CT images showed diffuse swelling of Waldeyer’s tonsillar ring, multiple enlarged lymphadenopathies, and splenomegaly. Acute EBV infection was confirmed considering clinical presentation and using the heterophile antibody, anti-nuclear and anti-viral capsid antigens, and quantitative PCR. The patient was managed with ventilatory support, empirical antibiotic therapy, and systemic corticosteroids, achieving a positive outcome. Our case illustrates the use of corticosteroids in managing severe upper airway obstruction complicating IM.

## Introduction

Infectious mononucleosis (IM) is usually a self-limited, benign lymphoproliferative disease caused by the Epstein-Barr virus (EBV). EBV is a gamma herpesvirus with a double-stranded DNA genome of about 172 kb [1]. In seroprevalence surveys, over 90% of adults worldwide are infected with this virus [1,2]. It most commonly affects the adolescent population, with a peak incidence in persons between 15 and 19 years (3.2-3.7 cases per 1000 persons) [1,3]. EBV is transmitted predominantly through infected saliva during kissing and less commonly through sexual contact, blood transfusions, or organ transplantations [1,2,4]. EBV can be spread using objects, such as a toothbrush or drinking glass, that an infected person recently used [4]. The most common clinical manifestations are sore throat, lymph node and tonsillar enlargement, fever, myalgia, and malaise [1,2,5]. Some complications of IM include concurrent *Streptococcus pyogenes* pharyngitis (30%), significant airway compromise (<5%), splenic rupture, and peritonsillar abscess (<1%) [2,5,6]. We describe a case of IM complicated by severe upper airway obstruction requiring ventilatory support and the use of corticosteroid therapy.

## Case presentation

An 18-year-old Hispanic man with a presumptive history of IM three years prior was transferred to our institution for the management of acute respiratory failure secondary to upper airway obstruction. Two days before the presentation, the patient complained of a progressive sore throat, tonsillar swelling, and difficulty speaking. The heterophile antibody test was checked and returned positive. Given the worsening of his condition and concerns for upper airway compromise, the patient was intubated for airway protection and administered a dose of intravenous dexamethasone before transference to our hospital.

On admission, vital signs were notable for a weight of 129 kg (BMI 35), temperature of 36.8 °C, heart rate of 94 beats/minute, blood pressure of 147/70 mmHg, respiratory rate of 18 breaths/minute, and oxygen saturation of 100% while on ventilatory support. The physical exam was notable for an enlarged neck and bilateral prominent and firm cervical lymphadenopathies. The endotracheal tube limited the oral exam. There was no evidence of skin abnormalities, including rash and jaundice. The abdominal exam was negative for evident organomegaly.

As seen in Table 1, initial laboratory results were remarkable for mild elevation of liver enzymes, a positive heterophile test, negative EBV viral capsid antigen (VCA) and EBNA antibodies (IgG), a positive EBV VCA IgM with an elevated titer, and positive EBV qualitative and quantitative PCR. There was no leukocytosis or lymphocytosis. However, few reactive lymphocytes were reported.

**Table 1:** Initial laboratory workup results. AST: aspartate aminotransferase; ALT: alanine aminotransferase; HIV: human immunodeficiency virus; CMV: cytomegalovirus; IgG: immunoglobulin G; IgM: immunoglobulin M; EBNA: Epstein-Barr nuclear antigen; EBV: Epstein-Barr virus; VCA: viral capsid antigen; PCR: polymerase chain reaction.

Tests	Results	Reference Range
White blood cell count	9.52 x 10^3^/µL	4.50-11.03 x 10^3^/µL
Lymphocytes	4.23 x 10^3^/µL	1.00-4.80 x 10^3^/µL
Reactive lymphocytes	Few	Negative
Red blood cell count	4.95 x 10^6^/µL	3.50-5.50 x 10^6^/µL
Hemoglobin	14.8 g/dL	12.0-15.0 g/dL
Platelets	124 x 10^3^/µL	150-450 x 10^3^/µL
Glucose	126 mg/dL	74-106 mg/dL
Blood urea nitrogen	10 mg/dL	7-17 mg/dL
Creatinine	0.80 mg/dL	0.66-1.25 mg/dL
Total bilirubin	0.8 mg/dL	0.2-1.3 mg/dL
AST	84 mg/dL	17-59 IU/L
ALT	134 mg/dL	21-72 IU/L
HIV 1/2 rapid 4th generation	Non-reactive	Non-reactive
CMV IgM	Negative	Negative
CMV IgG	Positive	Negative
Hepatitis B surface antigen	Negative	Negative
Hepatitis B core total antibody	Negative	Negative
Hepatitis B surface antibody	Positive	Negative
Hepatitis A IgM	Negative	Negative
Hepatitis C antibody	Negative	Negative
Heterophile test	Positive	Negative
EBNA IgG	Negative	Negative
EBV VCA IgG	Negative	Negative
EBV VCA IgM	Positive	Negative
EBV VCA IgM titer	>160.00 U/mL	<18.00 U/mL
EBV qualitative PCR	Positive	Negative
EBV quantitative PCR	2982 copies/mL	<200 copies/mL

**Figure 1:**
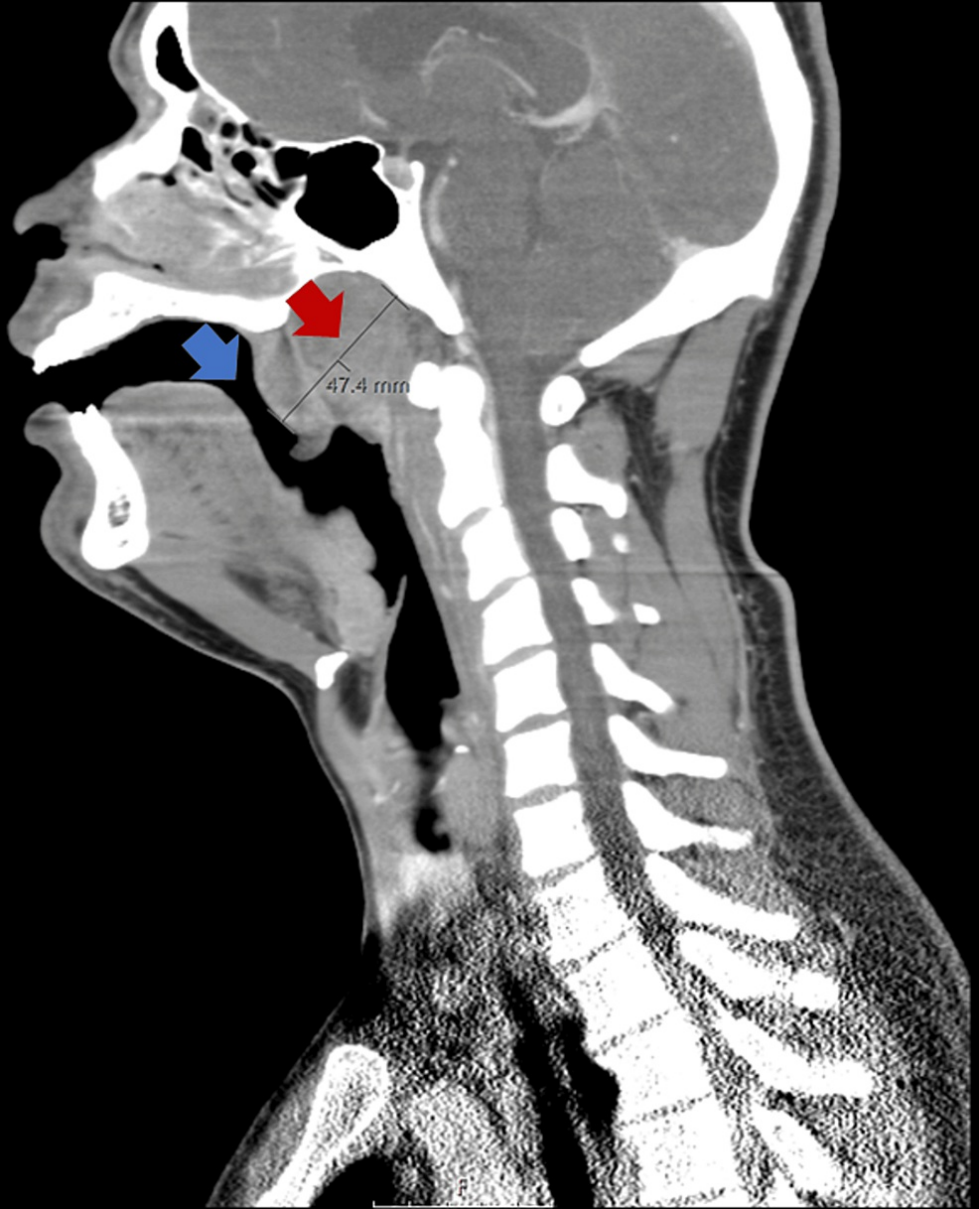
Neck CT scan sagittal view Diffuse swelling of Waldeyer’s tonsillar ring (red arrow). Nasopharyngeal airway narrowing (blue arrow).

**Figure 2:**
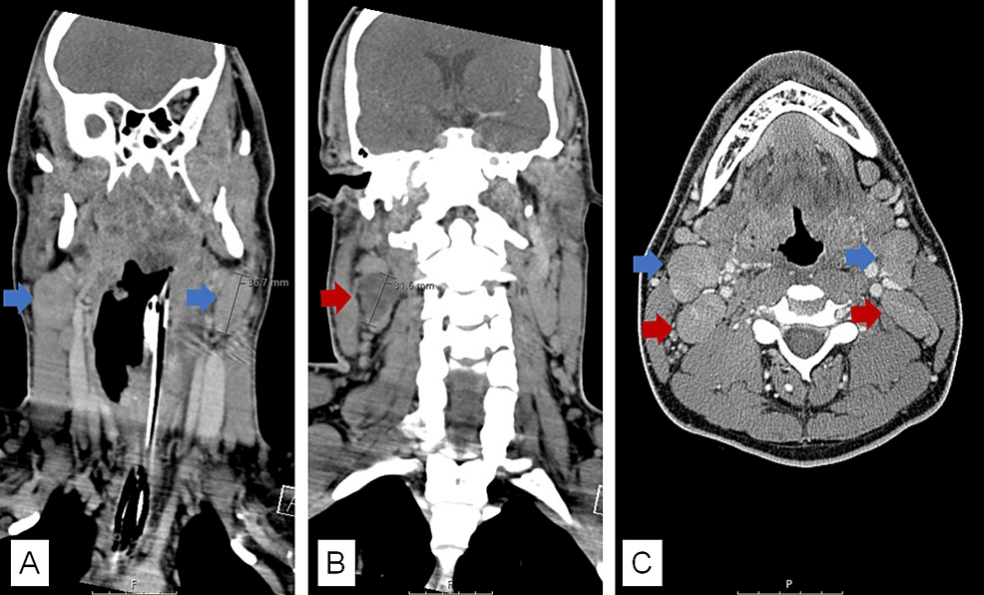
Neck CT scan coronal (A and B) and axial (C) views (A) Level 2A: bilateral cervical lymphadenopathies (blue arrows). (B) Level 2B: necrotic right cervical lymph node (red arrow). (C) Level 2A (blue arrows) and 2B (red arrows) cervical lymphadenopathies.

**Figure 3:**
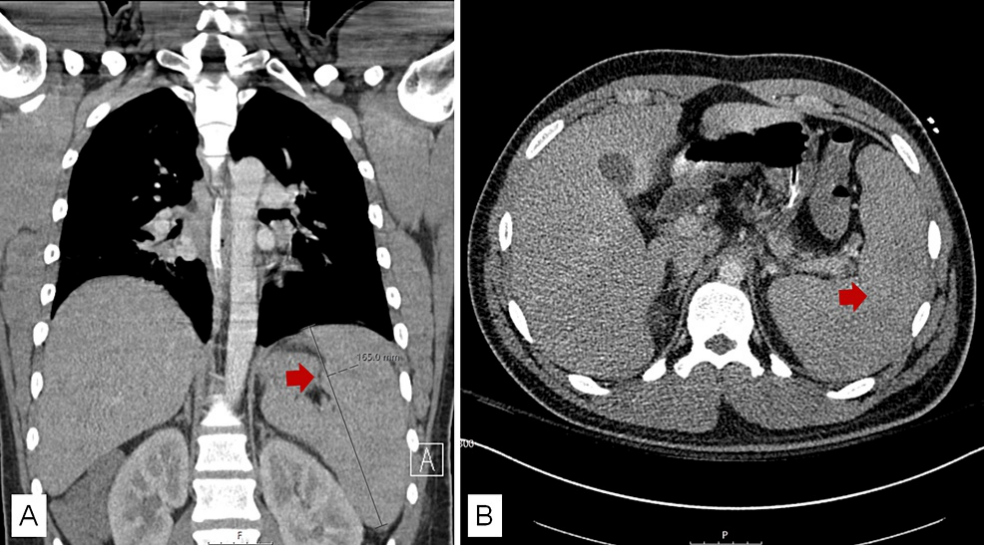
Abdomen CT scan coronal (A) and axial (B) views Splenomegaly (red arrow).

CT images showed diffuse swelling of Waldeyer’s tonsillar ring associated with severe narrowing of the nasopharyngeal airway producing obstruction (Figure 1) surrounded by bilateral reactive level 2 cervical lymphadenopathies and a necrotic right level 2B cervical lymph node (Figure 2). Splenomegaly (16.5 cm long in the coronal view) was another finding reported in CT images (Figure 3).

The patient was admitted to the medical ICU for ventilator management. The otolaryngology consultant, after evaluation, recommended against surgical intervention as no evident fluid collection(s) were seen in CT images, but empiric antibiotic coverage for potential bacterial superinfection and corticosteroid treatment. The patient received intravenous piperacillin-tazobactam and dexamethasone, respectively. The former was used as empiric therapy for possible bacterial superinfection, and the latter to help with the extensive inflammatory process in the airway. On day six, the anesthesia team took the patient to the operative room for a safe extubation, and it occurred without complications. An elongated palate was observed on examination, and potential obstructive sleep apnea features were noted. On day eight, after clinical improvement, the patient was discharged home with a short course of oral amoxicillin and prednisone. Extensive counseling to avoid contact sports for at least a month, the need for an outpatient sleep study, and a follow-up in the otolaryngology clinic were discussed.

## Discussion

While IM is generally a benign, self-limiting condition, it can cause severe otolaryngologic complications in up to 5% of patients [7]. Palatal and nasopharyngeal tonsil hypertrophy and edema of the surrounding soft tissue can lead to airway compromise and are among the most common indications for hospitalization. Younger children seem to be at greater risk [8] than adults. The likely risk factors contributing to airway obstruction in our patient included obesity (BMI 35), an elongated palate, and possible underlying obstructive sleep apnea. The cardinal symptoms of severe upper airway obstruction are stertor or stridor, dyspnea, intercostal and suprasternal retractions, tachypnea, and cyanosis, which demand emergent intervention. However, they might be absent until late in the disease process [9]. These symptoms were not reported in our case and were prevented by securing the airway.

IM should be suspected clinically in teenagers and young adults who present acutely ill with sore throat, cervical lymphadenopathy, fever, and fatigue [2]. The heterophile antibodies help support the diagnosis in suspected individuals. They are IgM-class antibodies directed against mammalian erythrocytes. They are not specific; therefore, false positive heterophile antibodies can be reported in conditions including other acute infections, autoimmune diseases, and cancer [2]. Given its non-specificity, false negative results can also be reported, especially in younger children [2,4]. Specific serologic tests can be performed when the diagnosis is uncertain, such as antibodies against EBV VCA and nuclear antigen (EBNA). EBV VCA IgM can be detected as early as a week before the onset of clinical symptoms [2,4,5]. EBV VCA IgG, on the other hand, can be detected within a month of illness [2,4,5]. Antibodies against EBNA develop within three months of illness. Therefore, if they are present during the acute disease, they rule out EBV acute infection [2,4,5]. Our patient had an acute EBV infection supported by a positive heterophile antibody test and VCA IgM, negative antibodies against EBNA, and the presence of EBV viremia.

The previous report of IM in our patient was likely a different acute illness, likely cytomegalovirus infection, due to the presence of a positive IgG and a negative IgM. Other conditions can have clinical manifestations similar to EBV, including cytomegalovirus, human herpesvirus 6, herpes simplex virus type 1, adenovirus, *Toxoplasma gondii, Streptococcus pyogenes*, and HIV acute infection [1,10]. Thus, they are considered heterophile-negative mononucleosis-like illnesses or mononucleosis syndrome [1,10].

Management of IM is primarily supportive; however, other strategies have been employed, such as corticosteroid and antiviral therapy [1,2]. The use of corticosteroids for managing IM remains controversial due to conflicting data and the possibility of impairing viral clearance or associated superinfections [1,2]. Its use is supported in cases of airway compromise, idiopathic thrombocytopenic purpura, or hemolytic anemia, but corticosteroids are not recommended in uncomplicated IM cases [1,2,11]. In a multicenter study conducted by Tynell et al., the combination of acyclovir and prednisolone did not affect the duration of symptoms or lead to an earlier return to school or work [12]. Wohl and Isaacson described their success in averting emergent surgical procedures using high-dose corticosteroids in children with compromised airways [13]. Some authors suggest acute tonsillectomy as a second-line treatment to treat upper airway obstruction that has failed to respond to corticosteroids. However, due to the high-risk perioperative bleeding (up to 13%), it is not strongly advocated [14,15].

Antivirals have also been used in the management of IM. Acyclovir and valacyclovir have *in-vivo* antiviral activity but have no clinical benefit [1,2]. Ganciclovir and valganciclovir are antivirals commonly used to manage EBV infection in immunocompromised patients, but no trials support their clinical efficacy [2]. Antiviral therapy cannot be the standard of care in managing IM until robust data are available from randomized controlled trials. Antivirals were not used in our patient.

## Conclusions

Upper airway obstruction is a rare but potentially lethal complication of IM in cases with rapid deterioration of respiratory condition if management is delayed. This highlights the importance of maintaining a high index of suspicion for airway compromise in high-risk individuals. Systemic corticosteroids should not be used in uncomplicated cases but should be considered in non-resolving and severe cases, and airway patency should be closely monitored. Endotracheal intubation may be required, as in the case we described. Refractory and severe cases may require a more invasive intervention, such as tonsillectomy or tracheostomy.

## References

[1] Luzuriaga K, Sullivan JL (2010). Infectious mononucleosis. N Engl J Med.

[2] Dunmire SK, Hogquist KA, Balfour HH (2015). Infectious mononucleosis. Epstein Barr Virus Volume 1.

[3] Chetham MM, Roberts KB (1991). Infectious mononucleosis in adolescents. Pediatr Ann.

[4] (2024). About Epstein-Barr virus (EBV). https://www.cdc.gov/epstein-barr/about-ebv.html.

[5] Ebell MH (2004). Epstein-Barr virus infectious mononucleosis. Am Fam Physician.

[6] Johnsen T, Katholm M, Stangerup SE (1984). Otolaryngological complications in infectious mononucleosis. J Laryngol Otol.

[7] Ganzel TM, Goldman JL, Padhya TA (1996). Otolaryngologic clinical patterns in pediatric infectious mononucleosis. Am J Otolaryngol.

[8] Jenson HB (2000). Acute complications of Epstein-Barr virus infectious mononucleosis. Curr Opin Pediatr.

[9] Kilham H, Gillis J, Benjamin B (1987). Severe upper airway obstruction. Pediatr Clin North Am.

[10] Hurt C, Tammaro D (2007). Diagnostic evaluation of mononucleosis-like illnesses. Am J Med.

[11] Thompson SK, Doerr TD, Hengerer AS (2005). Infectious mononucleosis and corticosteroids: management practices and outcomes. Arch Otolaryngol Head Neck Surg.

[12] Tynell E, Aurelius E, Brandell A (1996). Acyclovir and prednisolone treatment of acute infectious mononucleosis: a multicenter, double-blind, placebo-controlled study. J Infect Dis.

[13] Wohl DL, Isaacson JE (1995). Airway obstruction in children with infectious mononucleosis. Ear Nose Throat J.

[14] Glynn FJ, Mackle T, Kinsella J (2007). Upper airway obstruction in infectious mononucleosis. Eur J Emerg Med.

[15] Penman HG (1970). Fatal infectious mononucleosis: a critical review. J Clin Pathol.

